# Conspiracy thinking and the long historical shadow of Romanticism on authoritarian politics

**DOI:** 10.3389/fpsyg.2023.1185699

**Published:** 2023-10-03

**Authors:** Steven M. Smallpage, Robert L. Askew, Eric A. Kurlander, Joshua B. Rust

**Affiliations:** ^1^Department of Political Science, Stetson University, DeLand, FL, United States; ^2^Department of Psychology, Stetson University, DeLand, FL, United States; ^3^Department of History, Stetson University, DeLand, FL, United States; ^4^Department of Philosophy, Stetson University, DeLand, FL, United States

**Keywords:** Romanticism, conspiracy thinking, paranormal belief, pseudoscience, authoritarianism, social dominance, structural equation (SEM)

## Abstract

**Background:**

Similar effect sizes have been reported for the effects of conspiracy, pseudoscientific, and paranormal beliefs on authoritarian attitudes, which points to a conceptual problem at the heart of the conspiracy literature, namely lack of clarity as to what uniquely defines conspiracy beliefs and whether those unique elements contribute distinctly to authoritarian ideologies. To our knowledge, this is the first study to test empirically the predictive power of variance unique to each construct against covariance shared among these constructs when predicting authoritarian and anti-democratic attitudes.

**Methods:**

Online survey was administered to 314 participants in 2021 that included a battery of demographic and psychological measures. Hierarchical factor models were used to isolate unique variance from shared covariance among responses to items representing conspiracy, paranormal and pseudoscientific beliefs. Structural equation models were used to test their unique and shared effects on authoritarian and anti-democratic attitudes.

**Results:**

We found that our combined measurement model of paranormal thinking, conspiracism, and pseudoscience exhibited exceptional model fit, and that each construct was strongly predictive of both SDO and RWA (*r* = 0.73–0.86). Once the shared covariance was partitioned into a higher order factor, the residual uniqueness in each first order factors was either negatively related or unrelated to authoritarian and anti-democratic attitudes. Moreover, the higher order factor explained the gross majority of variance in conspiracy (*R*^2^ = 0.81) paranormal (*R*^2^ = 0.81) and pseudoscientific (*R*^2^ = 0.95) beliefs and was a far stronger predictor (*β* = 0.85, *p* < 0.01) of anti-democratic attitudes than political partisanship (*β* = 0.17, *p* < 0.01). Strong partisan identifiers of both parties showed much higher romanticism scores than party moderates.

**Conclusion and limitations:**

When predicting authoritarian and anti-democratic attitudes, we found no empirically unique contributions of conspiracy beliefs. Instead, we found that a shared factor, representing a ‘romantic’ mindset was the main predictor of authoritarian and anti-democratic attitudes. This finding potentially explains failures of interventions in stopping the spread of misinformation and conspiracy theories. Conspiracy theory researchers should refocus on the shared features that conspiracy thinking has with other unwarranted epistemic beliefs to better understand how to halt the spread of misinformation, conspiracy thinking, anti-science attitudes, and even global authoritarianism.

## Introduction

In 1932, a year before the Nazi seizure of power, the German lawyer and journalist Rudolf Olden published a collection of essays, *Prophets in the German Crisis: The Miraculous or the Enchanted*, by leading intellectuals regarding the role of conspiracism, pseudoscience, and paranormal thinking in generating the rise of rightwing authoritarianism (Olden, 1932). Politics was “an eternal struggle between rationality and the miraculous,” Olden observed. When “rationality comes under pressure in crisis; its weapons… are suddenly mute, it is eaten by doubt, it emigrates or is restricted” (Olden, 1932, p. 20). In the realm of “politics, the predominance of the miraculous forces out everyone that wants to think rationally” (Olden, 1932, p. 20). Millions of ordinary Germans, Olden lamented, sought salvation in “the occultists, who speak of unknown powers… that stream out of the Führer,” from “the parapsychologists…. to the proponents of esoteric sciences” (Olden, 1932, p. 18). Liberal intellectuals might want to dismiss these paranormal, conspiracist, and pseudoscientific affinities as a collective “neurosis” or psychosis, Olden concluded, but that would not make them any less real in building a psychological bridge to fascism (Olden, 1932, p. 20; Kurlander, 2017).

In the first decades of the 21st century, we have observed much of the same in the rise of QAnon, whose adherents have propagated, to take only a few examples, conspiracies accusing Hillary Clinton’s inner circle of child sex trafficking (i.e., Pizzagate), Hollywood liberals of participating in “blood-harvesting” akin to the antisemitic blood libel, and the Rothschilds of secretly deploying “space lasers” to create global warming. We have likewise witnessed an American President repeatedly warning his constituents about the “Deep State” and refusing to accept the outcome of the 2020 election on grounds of unsubstantiated voter fraud and the conspiracy-fueled January 6th march on the US Capitol that forced Congressional members to flee into safe rooms. All while there was growing resistance to public health measures, the wild spread of misinformation related to vaccines, and the erosion of trust in science at the height of a global COVID-19 pandemic.

The political ramifications of conspiracy thinking are difficult to ignore given our normative concern with the health of liberal democracy. One of the most consistent findings in the literature is that conspiracy thinking is positively associated with support for authoritarianism and anti-democratic beliefs and generally, though not always, right-wing authoritarianism and populism (Grzesiak-Feldman and Irzycka, 2009; Bruder et al., 2013; Miller et al., 2016; Richey, 2017; Federico et al., 2018; Golec de Zavala and Federico, 2018; Douglas et al., 2019; Enders and Smallpage, 2019; Wood and Gray, 2019; Kim and Kim, 2020; Dyrendal et al., 2021; Enders and Uscinski, 2021; Enders et al., 2022; Pilch et al., 2023). But conspiratorial thinking may not be unique as a habit of mind among authoritarian and anti-democratic populists. Highly related tendencies of thought like paranormal or supernatural thinking (Baker and Draper, 2010; Dyrendal et al., 2021; Rizeq et al., 2021) and anti-science and pseudoscientific beliefs (Landrum and Olshansky, 2019; Landrum et al., 2021) have been similarly implicated in the rise in authoritarian and anti-democratic attitudes.

However, when predicting authoritarian and anti-democratic attitudes the similarity in reported effect sizes between conspiracy, pseudoscientific, and paranormal beliefs points toward a conceptual problem at the heart of the conspiracy literature, namely, lack of clarity as to what uniquely defines conspiracy beliefs and whether those unique elements *distinctly* contribute to authoritarian ideologies (Goreis and Voracek, 2019; Pilch et al., 2023). Reviewing the literature from 2018 to 2021, Pilch et al. (2023) finds that there is still no consistent theory to tie together otherwise disparate findings in the literature. Conspiracy thinking is rooted in mistrust of authorities, but seemingly only liberal-procedural authorities as it was positively related to support for right-wing authoritarianism (Moscatelli et al., 2023). This poses a particularly grave problem when it comes to interventions to halt the spread of conspiracy thinking, misinformation, and anti-democratic beliefs, as it seems that we have not properly diagnosed the cause and may only be treating the symptoms (O’Mahony et al., 2023). In fact, addressing the antecedent habits of mind, often inoculating or priming non-affective, critical thinking has proven to be one of the only reliable ways of counteracting conspiracy thinking (O’Mahony et al., 2023). Conspiracy thinking, it seems, is a symptom of some other, deeper cause.

The correlation among conspiracy, pseudoscientific, paranormal beliefs and anti-democratic convictions has been periodically observed in the literature on conspiracy thinking. However, these empirical correlations may be due to a lack of conceptual clarity (Sutton and Douglas, 2023). Following Adorno, perhaps conspiracy thinking is a symptom of a particular narcissistic (“authoritarian”) personality that embraces a broad range of supernatural beliefs, and is consequently anti-democratic, prejudicial, and prone to fascism (Yendell and Herbert, 2022). Conspiracy thinking is also related to paranoia and distrust of officialdom, following the theory laid out by Hofstadter’s *Paranoid Style* (1964) (van der Linden et al., 2021). But conspiracy thinking is not simply tied to narcissism or any unique personality traits. Conspiracy thinking may be founded on “conspiracy intuitions,” i.e., a subjective, almost automatic response that things aren’t what they seem, or that one is being actively deceived by liberal political institutions, powerful corporations, and mainstream science (Risen and Roberts, 2022). This kind of subjective, automatic response is correlated positively with magical thinking and negatively with analytical thinking. Perhaps conspiracy thinking may even be beneficial to the believer, in that conspiracy narratives make “people feel important, help people rationalize their behavior and therefore make them feel legitimate, and have entertainment value by stimulating feelings of excitement” (van Prooijen 2022, p. 2). This individual-level ego-defensive disposition may also become a collective ego-defense, which in turn explains the relationship between conspiracy thinking and authoritarianism (van Prooijen, 2022). Of course, this explanation is not simply confined to conspiracy thinking, as “sensation seeking” is also correlated with supernatural beliefs (van Prooijen, 2022). It is difficult to see how believing in a conspiracy belief is different, on the one hand, from believing in supernatural and pseudo-scientific beliefs (Sutton and Douglas, 2023), and, on the other hand, how these beliefs in turn would motivate anti-democratic beliefs.

One way to make sense of the conceptual and empirical similarities among conspiracy thinking and other attitudes is to posit that the differences among conspiracy thinking, paranormal thinking, and beliefs in pseudoscience may only be superficial, i.e., that these “epistemically unwarranted beliefs” are best understood as aspects of a more general or shared idea complex (Lobato et al., 2014). This “relational” approach—which we endorse—mirrors the original studies by Theodor Adorno et al.’s *The Authoritarian Personality* and Richard Hofstadter’s *Paranoid Style*, which drew extensively on qualitative studies of the collapse of Weimar Germany and the rise of Nazism and saw the rise of these particular beliefs to be tied to a general mood or mindset (Stern, 1974; Hofstadter, 2008; Neumann, 2017; Adorno et al., 2019; Lowenthal and Guterman, 2021). For scholars like Adorno and Hofstadter, who referenced Lowenthal and Guterman, the interwar period saw a rise in an anti-Enlightenment— ‘romantic’—mindset prone to supernatural thinking, conspiracy thinking, a belief in pseudoscience, etc. which was curated by right-wing politicians and fringe groups and then operationalized toward illiberal ends. Conspiracy thinking is, on this view, one potential consequence of the “depersonalization and permanent insecurity of modern life” where one can imagine oneself “as a romantic defender of ancient traditions tramped down by modern industrialism” and is prone to “intermittent and unexpected acts of violence” lamenting the “disenchantment” of life, and one’s feeling that “something has gone astray” (Lowenthal and Guterman, 2021, p. 17). From this perspective, conspiracy thinking and conspiracy beliefs are not necessarily uniquely related to authoritarianism, but they are rather subdomains of a latent, shared “cognitive foundation” which we provisionally designate as a “romantic” mindset.

However, while the relationalist approach capitalizes on the shared empirical finding that conspiracy thinking is highly correlated with other types of “epistemically unwarranted beliefs,” it is important to note that these types of studies presume that conspiracy thinking does not have a *unique* effect on things like politically extreme attitudes, because it is not ontologically distinct from these other constructs. This relationalist approach is problematic for conspiracy theory research, which assumes that conspiracy theorizing has a unique effect on anti-democratic beliefs. One response to the relationalists is to argue that conspiracy thinking plays a unique and independent role from these other beliefs, but this is obscured by a lack of conceptual clarity on what uniquely defines conspiracy thinking and conspiracy beliefs relative to these others. Sutton and Douglas (2023) ask: “What, therefore, makes conspiracy theories special in this crowded field of strange and unhelpful beliefs” (Sutton and Douglas, 2023)? For Sutton and Douglas (2023) the goal is to define “logically” what constitutes a conspiracy theory, in hopes that this will clarify what “makes them ontologically different from other beliefs” (Sutton and Douglas, 2023). This “essentialist” approach to resolving the conceptual and empirical correlations among things like conspiracy thinking, paranormal thinking, and belief in pseudoscience tries to identify and thereby differentiate the essential and unique features of a conspiracy belief from other related constructs in the hope that conspiracy thinking will, contra the relationalists, uniquely explain anti-democratic beliefs.

Both the relationalist and essentialist approaches predict correlations between conspiracism and authoritarian attitudes; the critical distinction between them is whether conspiracism is *uniquely* predictive. The relationalist position posits that conspiracy thinking is functionally synonymous with other types of “epistemically unwarranted beliefs,” driven largely by a loosely related idea complex of intuition, distrust, and narcissism, on the one hand, and likely boredom and unease at the disenchantment with the world, on the other. But, following the essentialist position, it is possible that the correlations reported in the literature among these putatively distinct belief systems are merely methodological artifacts of poorly operationalizing conspiracy thinking and conspiracy beliefs relative to other types of beliefs. This is certainly an issue with Lobato et al. (2014), as their study predated the emergence of the more popular validated measures of these beliefs. We agree with essentialists that relationalist studies like Lobato et al. (2014) need to be updated to include validated measures of conspiracy thinking, pseudoscience beliefs, and paranormal thinking. However, it remains unclear whether stricter definitional criteria for what *uniquely* constitutes a conspiracy belief (i.e., the essentialist solution) would change or even clarify relationships between authoritarian attitudes and each of the putatively distinct conspiratorial, pseudo-scientific, and paranormal mindsets. Moreover, until empirical evidence is proffered that shows that scales like the Generic Conspiracy Belief Scale do not measure belief in conspiracy theories, we remain skeptical of the utility of stricter definitions that would undoubtedly stipulate away many items that are *de-facto* face valid as conspiracy beliefs. Nevertheless, the essentialist position, irrespective of the need for stricter definitions, posits that at least some non-trivial covariance between conspiracy beliefs and authoritarian attitudes is *uniquely* attributable to conspiracy beliefs. Yet we know of no empirical studies to date that have tested the predictive power of the unique and shared covariance between conspiracy thinking and authoritarian and anti-democratic attitudes.

Hence, following relationalist theories proposed by Adorno et al. and others who suggest that conspiracy, paranormal, and pseudoscientific thinking “shares an underlying cognitive foundation”— what we are tentatively calling “romanticism” for the purposes of this paper— we would expect to find strong evidence for the predictive power of the shared factor on authoritarian beliefs. Following the essentialists, we would expect to find a unique predictive relationship between conspiracy thinking and anti-democratic beliefs—even when controlling for what is shared. As such, the purpose of this present project is to isolate and separate the shared and unique covariance among conspiracy thinking, paranormal thinking, and beliefs in pseudoscience to compare directly the relative predictive force of these constructs on authoritarian and anti-democratic beliefs. By parsing the shared and unique empirical relationships between conspiracy thinking and anti-democratic beliefs we gain a clearer understanding of the ontology of the psychological structures and motivators of conspiracy thinking, and the extent to which conspiracy thinking uniquely predicts anti-democratic beliefs. To that end, the aims of the current study are to interrogate the uniqueness and distinctiveness of each of these constructs and to explore their distinctive and collective contributions to anti-democratic and authoritarian attitudes through the following research questions:

Question 1: To what degree do measures of paranormal thinking, pseudoscience, and conspiracism, cohere as distinct factors and how strongly do they individually predict anti-democratic attitudes?

Question 2: How interrelated are the constructs of paranormal thinking, pseudoscience, and conspiracism and is the strength of intercorrelations sufficiently strong for a higher order factor of romanticism to emerge and animate each construct?

Question 3: Are anti-democratic attitudes better predicted by the emergent higher-order factor of romanticism or by the residual uniqueness in each sub-factor of paranormal thinking, pseudoscience, and conspiracism?

Question 4: What is the functional form of the relationship between romanticism and anti-democratic attitudes and is it consistent across political identities?

Question 5: How does the relative effect of romanticism compare to the relative effect of political identities when predicting anti-democratic attitudes?

Question 6: Do socio-demographic and dispositional factors correlate with romanticism in theoretically informative ways?

## Methods

### Participants

We administered a large survey to an internet sample of 314 participants. Table 1 presents a demographic profile of our sample, which predominantly self-reported as white (73%), male (62%), younger (52% aged 18–34), college educated (89% with bachelor’s degrees), Democratic leaning (67%) and employed full time (89%).

**Table 1:** Demographic profile of the study sample.

Characteristic	*n* = 314	%
Age		
18–24	16	5.1
25–34	146	46.5
35–44	77	24.5
45–54	52	16.6
55–64	18	5.7
65 or older	5	1.6
Race		
White, non-hispanic	228	72.6
Black, non-hispanic	37	11.8
Hispanic	25	8.0
Other, non-hispanic	24	7.6
Gender: female	120	38.2
Education		
High school diploma or equivalent (GED)	4	1.3
Some college but no degree	30	9.6
Bachelor’s degree (BA)	194	61.8
Graduate degree (MA, MS, MD, PhD)	86	27.4
Employment		
Employed full time	278	88.5
Employed part time	17	5.4
Unemployed	8	2.5
Retired/Disabled/Student	11	3.5
Political identity		
Strong democratic	153	48.7
Somewhat democratic	38	12.1
Leans democratic	21	6.7
Neither	13	4.1
Leans republican	12	3.8
Somewhat republican	18	5.7
Strong republican	59	18.8
Military or veteran	105	33.4
Religious affiliation (missing: *n* = 7)		
Christian, mainline protestant	44	14.3
Christian, black protestant	16	5.2
Christian, evangelical protestant	17	5.5
Christian, Catholic (Roman Catholic)	164	53.4
Jewish	8	2.6
Non-Christian/Non-Jewish/Other not listed	8	2.6
Atheist, agnostic, none	39	12.7
Spiritual, but not religious	11	3.6
Religious service attendance (missing: *n* = 2)		
Never	54	17.3
A few times a year	41	13.1
Once or twice a month	66	21.2
Almost every week	65	20.8
Every week	86	27.6

### Procedure and measures

The survey was administered April 26th to April 27th 2021 via Amazon’s Mechanical Turk. There were four attention checks throughout the survey. Of the 443 respondents that attempted our survey, 40 respondents did not complete the survey, and 89 failed at least one attention check. These 129 respondents were excluded from the analysis. The survey included a variety of questions related to demographics, political attitudes and intuitions, and scales widely used in the political scientific literature to measure paranormal thinking, pseudoscientific beliefs, conspiracism, rightwing authoritarianism and social dominance orientation, as follows.

Paranormal beliefs were measured according to the Revised Paranormal Beliefs Scale (RPB) (Tobacyk, 2004). This scale is composed of 26 items sampled from 7 content domains of traditional religious belief, psi, witchcraft, superstition, spiritualism, extraordinary life forms, and precognition. Response options ranged from 1 (Strongly Disagree) to 7 (Strongly Agree). Although adequate psychometric properties have been reported for a bifactor model of scale items, others have reported conceptual and psychometric evidence suggesting fewer dimensions among the items is warranted (Thalbourne et al., 1995; Lange et al., 2000; Rizeq et al., 2021). Given the psychometric evidence in the current study (detailed below) we modeled these items as unidimensional.

Pseudoscientific beliefs were measured with the Pseudoscientific Beliefs Scale (PBS) (Fasce, 2017). This scale is composed of 30 items sampled from content domains representing both pseudoscientific-theory promotion and science denialism. Response options ranged from 1 (Strongly Disagree) to 5 (Strongly Agree). Given the psychometric performance of items in the current study and recent evidence from others suggesting a unidimensional factor structure (Fasce, 2017) we modeled these items as a single factor.

Conspiracy beliefs were measured with the Generic Conspiracy Beliefs Scale (GCB) (Brotherton et al., 2013). This scale is composed of 15 items sampled from content domains representing government malfeasance, extraterrestrial cover up, malevolent globalism, among others. Response options ranged from 1 (Strongly Disagree) to 5 (Strongly Agree). While the GCB has psychometric evidence supporting the use of 5 correlated factors, given the recommendation of authors of the scale and the improved psychometric performance of items when modeled with a unidimensional factor structure, we modeled these items as a single factor (Brotherton et al., 2013).

Right-Wing Authoritarianism (RWA) was measured with the Right-Wing Authoritarianism Scale (Johnson, 2020; Altemeyer, 2020; Altemeyer, 1983; Manganelli Rattazzi et al., 2007). Response options for this scale range from 1 (Very Strongly Disagree) to 9 (Very Strongly Agree). The scale is composed of 22 items sampled from content domains of authoritarian aggression, authoritarian submission, and conventionalism, and while some have proposed modeling the domains as 3 distinct factors, this scale has traditionally been modeled as unidimensional (Funke, 2005). As others have observed, fit indices for the unidimensional model were undermined by substantial method variance among the negatively worded items, and as such, only the positively worded items were utilized (Manganelli Rattazzi et al., 2007). Nevertheless, as a sensitivity check, we assessed the correlation among scores using only the positively worded items and scores using all positively and negatively worded items, and a correlation of 0.98 indicated that the negligible loss in validity was more than made up for with improved model fit.

Social Dominance Orientation (SDO) was measured with the Social Dominance Scale (Pratto et al., 1994). This scale is composed of 14 items with response options ranging from 1 (Strongly Disagree) to 5 (Strongly Agree). While the developers of the scale provided strong conceptual and psychometric evidence in terms of reliability and validity of scale scores, the support for a unidimensional structure was weak. Follow up studies (Jost and Thompson, 2000; Ho et al., 2012) suggested the 2 factor solution (i.e., passive equality and aggressive inequality) was optimal. Given our stated hypotheses, we focused our analysis on the aggressive inequality factor.

### Statistical analysis

Because of the diversity of methods employed, we detail each set of hypothesis tests by research question below. All data preparation and statistical hypothesis tests were carried out using R Studio (99), R statistical software (R Core Team, 2022) the tidyverse (Wickham et al., 2019), readstata13 (Garbuszus and Jeworutzki, 2021), expss (Demin, 2022), psych (Revelle, 2022), emmeans (Lenth, 2022), semPlot (Epskamp, 2022), and lavaan (Rosseel, 2012) packages. This study was approved by the Institutional Review Board.

Question 1: *To what degree do measures of paranormal thinking, pseudoscience, and conspiracism, cohere as distinct factors and how strongly do they individually predict anti-democratic attitudes?* To answer this question, we modeled responses to survey items for each scale using Confirmatory Factor Analysis (CFA). CFA is an analytical approach to the measurement of latent constructs that assumes that each item on a survey serves as an imperfect measure of the construct of interest. As such, factor analysis mathematically separates differences that are shared among the survey items (representing real differences in the construct) from differences that are unique to each item (representing measurement error). Because the surveys employed Likert response scales, we factor analyzed polychoric correlation matrices using diagonally weighted least squares (WLSMV), which is a more robust method for ordinal response data (Holgado–Tello et al., 2010; Li, 2016).

As a preliminary first step, the psychometric structure of each scale was evaluated in isolation. Separate evaluations were carried out for measures of SDO, RWA, GCB, RPB, and PBS, all of which were directly related to our primary study hypotheses. However, given our interests in describing the variable Romanticism in post-hoc exploratory analysis, we also attempted (unsuccessfully) to find psychometrically robust scales of esthetic, rational, and experiential intuitions. Nevertheless, we were able to find a handful of items in the literature (Pacini and Epstein, 1999) that were face valid for esthetic (e.g., “It is important that I feel inspired everyday”), rational (e.g., “I enjoy solving problems that require hard thinking”), and experiential (e.g., “Using my gut feelings usually works well for me in figuring out problems in my life”) intuitions that achieved adequate reliability and structural validity for post-hoc exploratory analysis (see Appendix A).

The performance of each measure was evaluated using traditional indicators of statistical model fit, such as the Comparative Fit Index (CFI) and the Tucker Lewis Index (TLI), with values >0.90 indicating adequate model fit and values >0.95 indicating exceptional fit (Hu and Bentler, 1999). We also evaluated model mis-fit using the Standardized Root Mean Square Residual (SRMR) and the Root Mean Square Error of Approximation (RMSEA). In terms of mis-fit, values <0.08 are considered indicators of acceptable model mis-fit, and values <0.05 are considered exceptional (Hu and Bentler, 1999). When individual survey measures failed to achieve adequate model fit or when estimation led to non-invertible information matrices, we adjusted the model better to accommodate the correlational structure among item responses. For example, items were dropped from several scales that provided substantially more noise than information (i.e., when individual items were too weakly correlated with the latent constructs; when *λ* < 0.40) in order to achieve adequate model fit and precision in measurement. When putatively multi-dimensional scales yielded subscale scores that were correlated with each other at 0.90 or higher, those scales were modeled as unidimensional measures. Multiple scales employed in this study required at least minor adjustments to achieve adequate fit; a complete table of included and excluded items, factor loadings, and model fit indices for all scales used in this study are provided in Appendix A.

**Figure 1:**
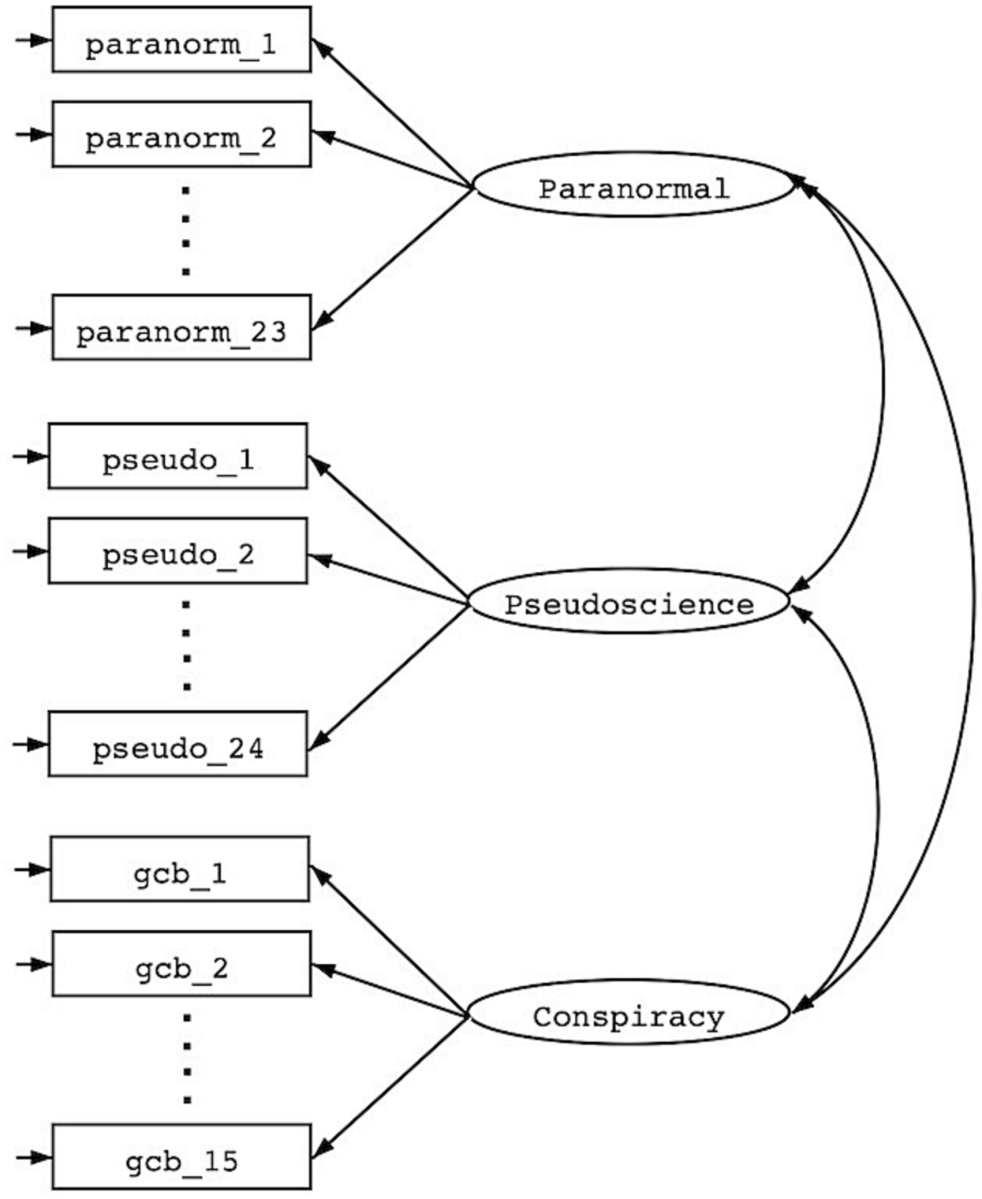
Measurement model of 3 correlated factors: paranormal, pseudoscience, and conspiracy beliefs.

After each scale was refined in isolation, we then examined the fit of a unified measurement model. Figure 1 is a graphical representation of a (correlated) 3-factor measurement model of Paranormal thinking, Pseudoscientific beliefs, and Conspiracism (Generic Conspiracy Beliefs). This measurement model implies that differences in responses to survey items are a function of both individual differences in traits of respondents modeled as latent factors (i.e., putatively paranormal thinking, pseudoscience, and conspiracism on the right-hand side of the figure) and differences attributable to item-specific measurement error (represented by → on the left-hand side of the figure). After assessing the proportion of individual differences across all survey items and all respondents that were accounted for by the latent variables, we then compared the magnitude of correlations among each of the factors with measures of SDO and RWA.

**Figure 2:**
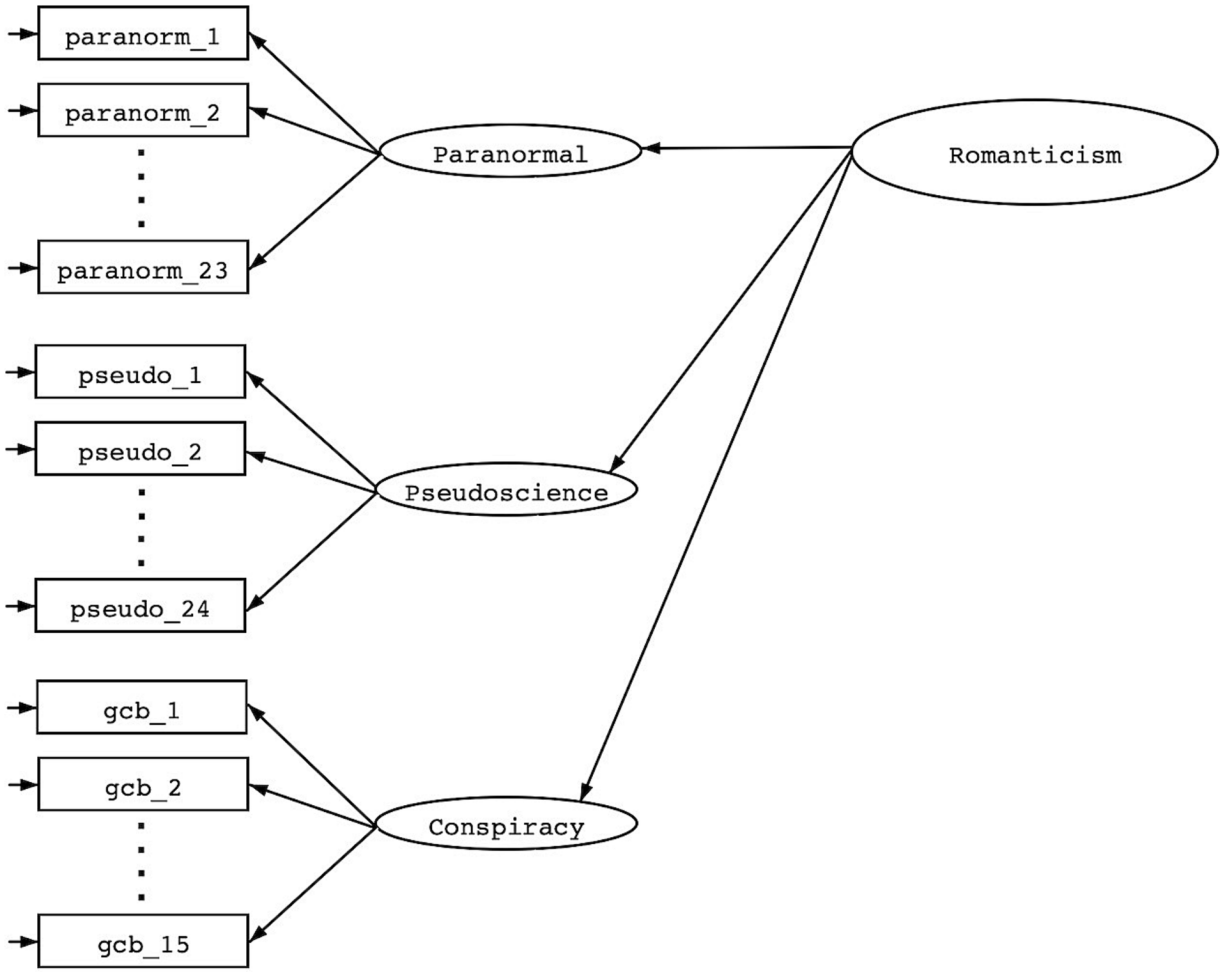
Hierarchical measurement model of “Romanticism.”

Question 2: *How interrelated are the constructs of paranormal thinking, pseudoscience, and conspiracism and is the strength of intercorrelations sufficiently strong for a higher order factor of romanticism to emerge and animate each construct*? To answer this question, we constructed a hierarchical factor model with one second-order factor and three first-order subordinate factors (See Figure 2). This measurement model implies that individual trait differences in the higher-order factor (i.e., Romanticism) drive individual trait differences in the three subordinate factors (i.e., paranormal thinking, pseudoscience, and conspiracism), that in turn drive differences in responses to individual survey items. We then examined the factor loadings to evaluate how well paranormal thinking, pseudoscience, and conspiracism served as indicators of the broader construct of Romanticism. We also assessed the relative performance of the hierarchical model using the same statistical indices of model fit and mis-fit.

**Figure 3:**
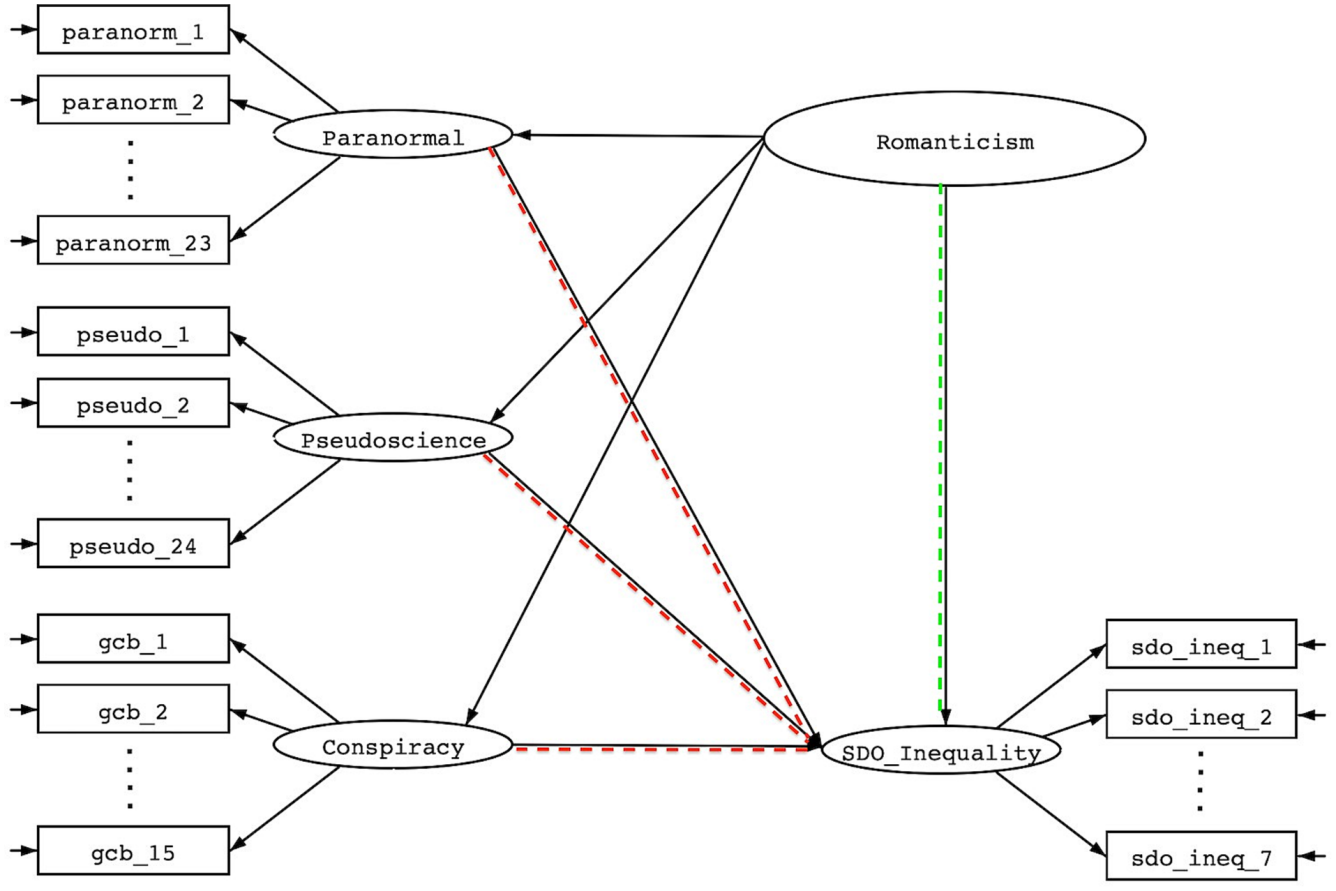
Structural equation model enabling testing of relative effects of Romanticism and residual uniqueness of paranormal thinking, pseudoscience, and conspiracism on social dominance orientation.

Question 3: *Are anti-democratic attitudes better predicted by the emergent higher-order factor of romanticism or by the residual uniqueness in each sub-factor of paranormal thinking, pseudoscience, and conspiracism?* To answer this question, we used structural equation models to simultaneously predict SDO with factor scores representing Romanticism and factor scores representing variability exclusively attributable to the individual domains of paranormal thinking, pseudoscience, and conspiracism (See Figure 3). This model facilitates direct testing of whether Romanticism (dotted arrow on the right) is the main driver of SDO or whether there are unique elements of paranormal thinking, pseudoscience, and conspiracism (three dotted arrows on the left) that are predictive of SDO. We then followed the same procedure for RWA to compare relative effects. Adequacy of our models were again evaluated using traditional statistical indices of model fit and mis-fit.

Question 4: *What is the functional form of the relationship between romanticism and anti-democratic attitudes and is it consistent across political identities?* To answer this question, we created scatterplots with Locally Estimated Scatterplot Smoothing (i.e., LOESS curves) and compared the curves between subgroups defined by party identification.

Question 5: *How does the relative effect of romanticism compare to the relative effect of political identities when predicting anti-democratic attitudes?* In order to answer this question, we used a structural equation model that simultaneously tested effects of Romanticism and party affiliation on SDO and RWA.

Question 6: *Do socio-demographic and dispositional factors correlate with romanticism in theoretically informative ways?* To answer this question, we examined correlations between scores representing Romanticism with scores from *ad-hoc* measures of esthetic, rational, and experiential intuitions. We also evaluated differences in Romanticism scores among groups defined by multiple demographic indicators using *F* tests.

## Results

Our results were very promising, suggesting that there is a coherent dimension of contemporary American public opinion comprising aspects of conspiracy thinking, paranormal beliefs, and pseudoscientific beliefs that strongly predicts authoritarianism across partisanship, which we have called Romanticism. With respect to Question 1, we found that our combined measurement model of paranormal thinking, conspiracism, and pseudoscience exhibited exceptional model fit (robust CFI = 0.97, TLI = 0.97, SRMR = 0.06, RMSEA = 0.03) and that each construct was strongly predictive of both SDO and RWA (*r* = 0.73–0.86). With respect to Question 2, we found that these putatively unique factors were sufficiently highly correlated with each other to warrant specification of a higher order factor which we identify as Romanticism. In fact, each of the first order factors loaded more highly onto the superordinate factor (*λs* ≥ 0.90) than the individual items loaded on their respective first order factors (0.88 ≥ *λs* ≥ 0.40). Moreover, the gross majority of variance in paranormal thinking (*R*^2^ = 0.81), conspiracism (*R*^2^ = 0.81), and pseudoscience (*R*^2^ = 0.95) was shared and explained by Romanticism, with marginal uniqueness remaining as residual variance. This hierarchical factor model demonstrated excellent statistical fit by all available indices (robust CFI = 0.97, TLI = 0.97, SRMR = 0.06, RMSEA = 0.03).

**Figure 4:**
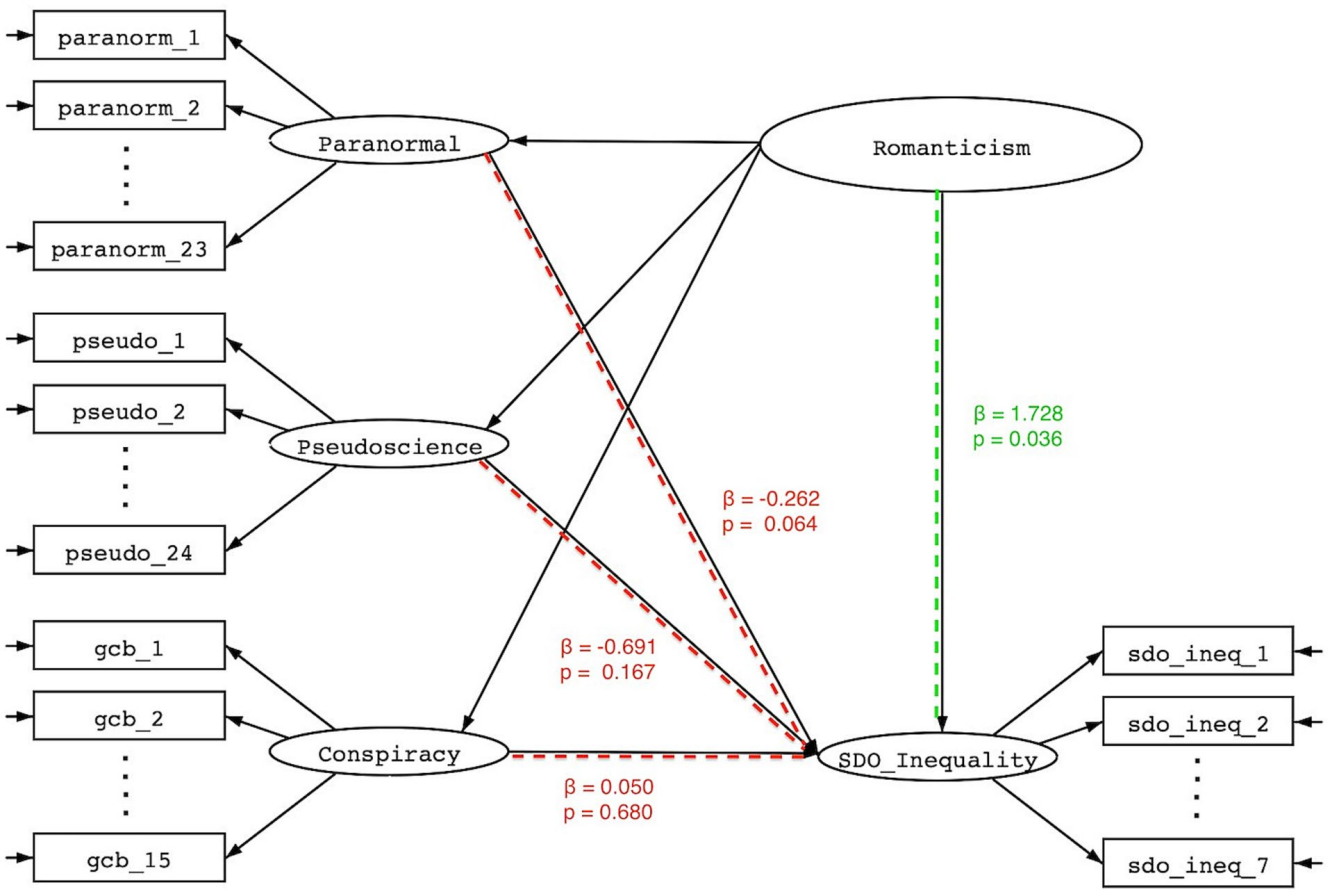
Standardized effects of Romanticism and residual uniqueness of paranormal thinking, pseudoscience, and conspiracism on social dominance orientation.

With respect to Question 3, we found that the higher order factor of Romanticism was far more effective at predicting SDO than the unique features of paranormal thinking, conspiracism, and pseudoscience (See Figure 4). In fact, once the shared correlation among these subfactors was partitioned into the superordinate factor of Romanticism, the remaining differences in paranormal thinking, conspiracism, and pseudoscience were all negatively related or unrelated to both SDO (*β*= − 0.26, *p* = 0.06; *β*=0.05, *p* = 0.68; *β*= − 0.69, *p* = 0.17, respectively) and RWA (*β*= − 0.15, *p* = 0.27; *β*= − 0.14, *p* = 0.29; *β*= − 0.95, *p* = 0.22, respectively).

**Figure 5:**
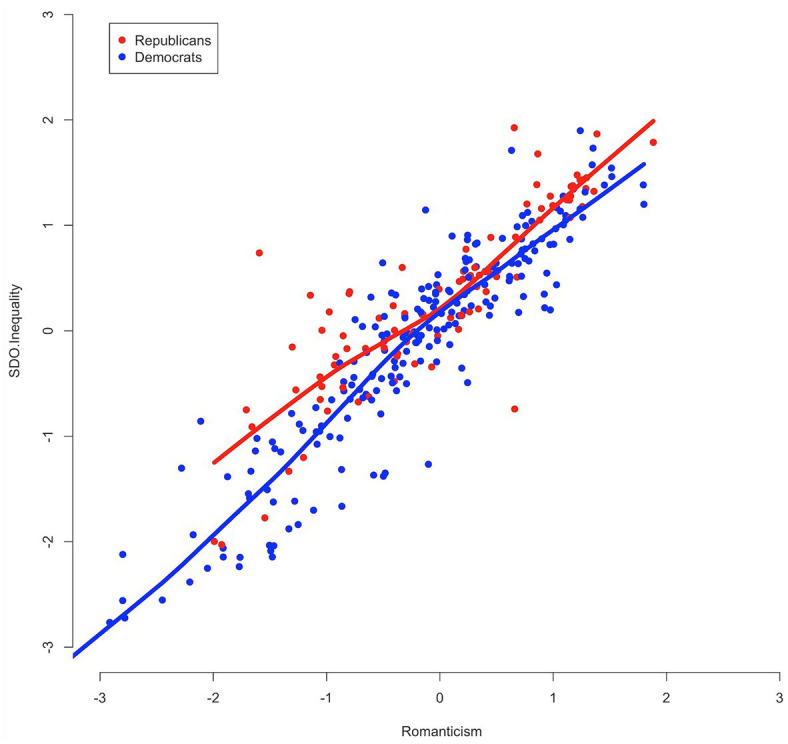
Scatterplot of Romanticism and social dominance orientation (Inequality) scores with LOESS curves, by partisanship.

**Figure 6:**
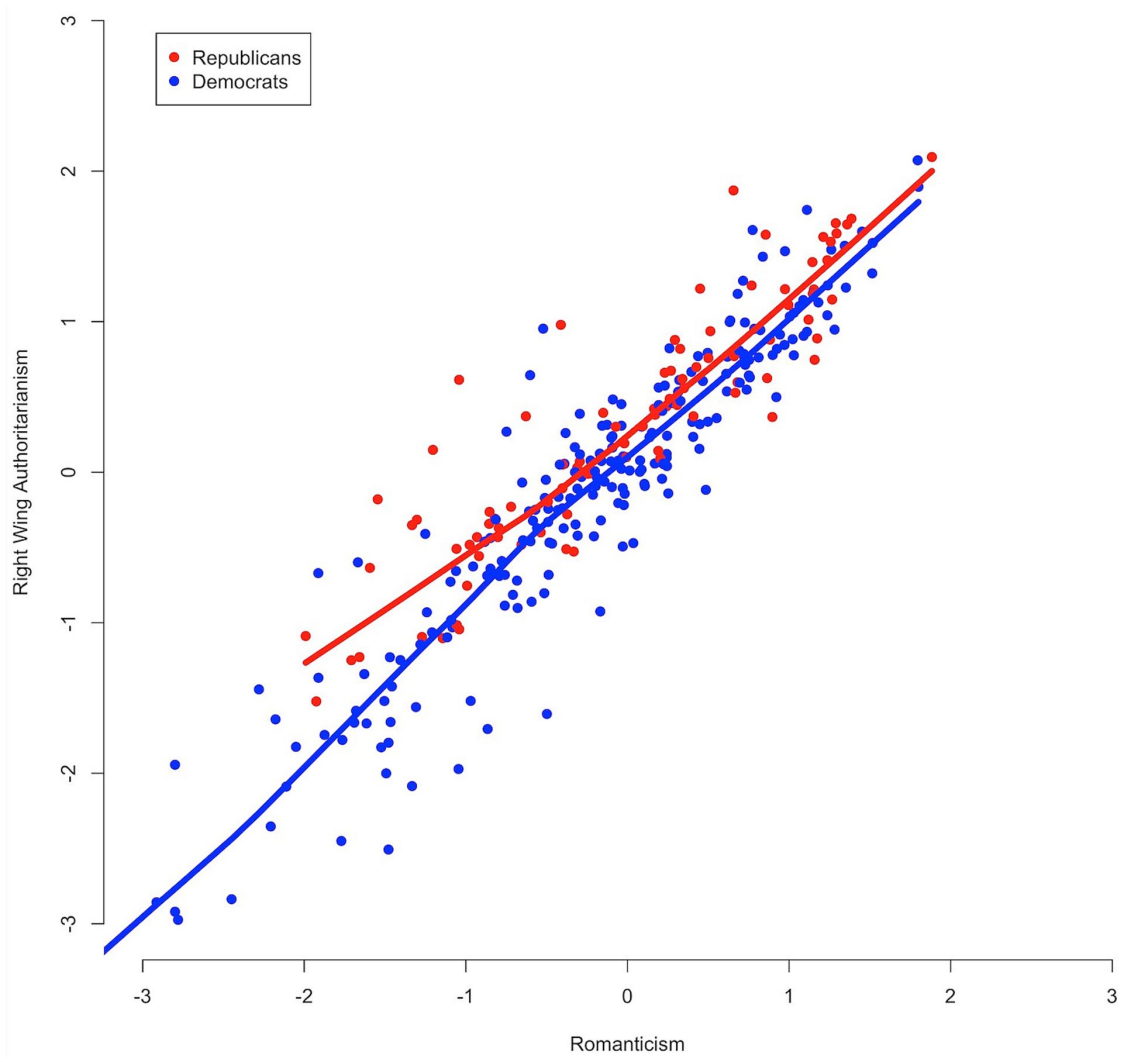
Scatterplot of Romanticism and right-wing authoritarianism scores with LOESS curves, by partisanship.

With respect to Question 4, we assessed the functional form of associations between Romanticism and SDO and RWA through LOESS regressions. The curves in Figure 5 indicate an approximately linear relationship between Romanticism scores (higher scores are more romantic) and SDO scores (higher scores are more socially dominant), and this linear relationship appears consistent across groups defined by party identification (blue for respondents self-identifying as Democrat and red for respondents self-identifying as Republican). A similar functional form is observed in Figure 6 with respect to the relationship between Romanticism and RWA.

With respect to Question 5, we found that the effect of Romanticism (*β*=0.85, *p* < 0.01) on SDO was much larger than the effect of political identity (Republican vs. Democrat: *β*=0.17, *p* < 0.01) with the gross majority of the variance in SDO explained by these predictors (multiple *R^2^* = 0.75). Likewise, the effect of Romanticism (*β*=0.88, *p* < 0.01) on RWA was much larger than the effect of political identity (Republican vs. Democrat: *β*=0.18, *p* < 0.01) with the gross majority of the variance in RWA scores explained by the model (multiple *R^2^* = 0.80).

With respect to Question 6, Romanticism scores correlated with scores representing esthetic (*r* = 0.72, *p* < 0.01), rational (*r* = −0.30, *p* < 0.01), and experiential (*r* = 0.69, *p* < 0.01) intuitions in theoretically expected ways. We also found interesting differences in levels of Romanticism by multiple socio-demographic indicators, including race, education, employment, military service, and religious affiliation, as illustrated in Table 2. As per conventions for model identification, the mean Romanticism score was constrained to 0.00 and the standard deviation to 1.00. Given that multiple subgroups in our sample were too small to support convincing generalizability claims regarding the population prevalence of Romanticism, we instead focused our analysis on how well general trends among subgroups of our sample mapped onto theory, only reporting provisional *F* tests for reference (as opposed to all pair-wise comparisons of means). While age and gender were not statistically associated with Romanticism, race, education, and employment were all highly significant (*p* < 0.001). Higher scores were observed for Black (*M* = 0.16, SE = 0.15) and Hispanic (*M* = 0.14, SE = 0.19) participants as well as for those with bachelor’s degrees (*M* = 0.13, SE = 0.07) and full-time employment (*M* = 0.08, SE = 0.06). Participants who were veterans or currently serving in the military (*M* = 0.47, SE = 0.09) were much higher in Romanticism than those with no military experiences (*M* = −0.27, SE = 0.06). With respect to religious affiliation, the lowest levels of Romanticism were observed for atheists and agnostics (*M* = −1.34, SE = 0.13) and the highest levels were observed for mainline Protestant Christians (*M* = 0.30, SE = 0.13) and Roman Catholics (*M* = 0.21, SE = 0.06). Likewise, a consistently positive incremental effect was observed in religious service attendance from “never” (*M* = −1.12, SE = 0.11) to “every week” (*M* = 0.42, SE = 0.09). Lastly, political identity was strongly associated with levels of Romanticism with the highest levels observed for those identifying as strongly Republican (*M* = 0.42, SE = 0.12) or strongly Democratic (*M* = 0.16, SE = 0.07).

**Table 2:** Differences in levels of Romanticism among demographic subgroups.

Characteristic	*M*	SE	*p* (*F* test)
Age			0.33
18–24	−0.09	0.25	
25–34	−0.12	0.08	
35–44	0.15	0.11	
45–54	0.09	0.14	
55–64	−0.13	0.23	
65 or older	−0.42	0.44	
Race			<0.01
White, non-hispanic	0.05	0.06	
Black, non-hispanic	0.16	0.15	
Hispanic	0.14	0.19	
Other, non-hispanic	−1.18	0.19	
Gender			0.55
Female	−0.07	0.09	
Male	0.00	0.07	
Education			<0.01
High school diploma or equivalent (GED)	−0.88	0.46	
Some college but no degree	−0.97	0.17	
Bachelor’s degree (BA)	0.13	0.07	
Graduate degree (MA, MS, MD, PhD)	0.01	0.10	
Employment			<0.01
Employed full time	0.08	0.06	
Employed part time	−0.43	0.22	
Unemployed looking for work	−0.85	0.41	
Unemployed not looking for work	−1.19	0.53	
Retired	−2.21	0.53	
Student	−1.03	0.35	
Disabled	−1.68	0.93	
Political identity			<0.01
Strong democratic	0.16	0.07	
Somewhat democratic	−0.65	0.15	
Leans democratic	−0.72	0.20	
Neither	−0.77	0.25	
Leans republican	0.01	0.26	
Somewhat republican	−0.38	0.21	
Strong republican	0.42	0.12	
Military or veteran	0.47	0.09	<0.01
Non-military or veteran	−0.27	0.06	
Religious affiliation (missing: *n* = 7)			<0.01
Christian, mainline protestant	0.30	0.13	
Christian, black protestant	0.20	0.21	
Christian, evangelical protestant	−0.31	0.20	
Christian, Catholic (Roman Catholic)	0.21	0.06	
Jewish	0.19	0.29	
Non-Christian/Non-Jewish/Other not listed	0.07	0.29	
Atheist, agnostic, none	−1.34	0.13	
Spiritual, but not religious	−0.73	0.25	
Religious service attendance (missing: *n* = 2)			<0.01
Never	−1.12	0.11	
A few times a year	−0.43	0.13	
Once or twice a month	0.16	0.10	
Almost every week	0.36	0.10	
Every week	0.42	0.09	

## Discussion

This paper set out to empirically test the differential predictive strength of the unique variance of conspiracy thinking, paranormal thinking, and beliefs in pseudoscience relative to their shared covariance when predicting authoritarian attitudes. First, we replicated the longstanding finding of the literature that the extant validated, commonly used scales of conspiracy thinking, paranormal thinking, and beliefs in pseudoscience are each individually predictive of authoritarian attitudes. Second, we replicated the findings of Lobato et al. (2014), but using validated scales, showing that the correlations among these constructs are sufficiently strong and that we have good reason to believe there is a higher order factor that animates each of the three. Third, for the first time in extant literature on conspiracy thinking, we examined the differential predictive strength of each of the unique constructs and the shared construct on authoritarianism. After controlling for the shared correlation, we found that conspiracy thinking is not a significant predictor of authoritarian attitudes (nor are paranormal beliefs or beliefs in pseudoscience). Having demonstrated that there is a meaningful shared dimension predicting anti-democratic beliefs, we turned toward exploring the nature of this dimension. Fourth, we demonstrated a linear relationship between the shared dimension and anti-democratic beliefs for both Democrats and Republicans. Fifth, we found that the shared dimension was a stronger predictor of authoritarian attitudes than partisanship. Finally, we provided preliminary correlational evidence to help outline the contours of the shared dimension.

These findings have several implications for the theoretical and empirical study of conspiracy thinking. The study of conspiracy thinking has been moored by conceptual and measurement debates (Goertzel, 1994; Brotherton et al., 2013; Bruder et al., 2013; Oliver and Wood, 2018; Douglas et al., 2019; Enders and Smallpage, 2019; Uscinski and Enders, 2022). Our paper now adds a fine point on the need for researchers to grapple with the ontological problem that Sutton and Douglas (2023) raised: what makes conspiracy thinking unique? We find that conspiracy thinking is not, as currently measured, empirically distinguished from other constructs, like paranormal thinking and belief in pseudoscience. One might, as Sutton and Douglas (2023) do, think that the problem lies in the fact that these constructs are not sufficiently conceptually distinguished. This is not our conclusion. Though perhaps further research on the alignment between Sutton and Douglas’ (2023) features and the GCB Scale may prove fruitful, we are confident that the items that constitute the GCB Scale reasonably trace conspiracy thinking. Therefore, following the relationalist position described above, we think the correlations among conspiracy thinking, paranormal thinking, and beliefs in pseudoscience point to the need to get clearer about how these constructs might be related to each other through a set of shared features.

What might characterize the shared variable we uncovered? In what follows, we consider several potential candidates: anti-establishment orientations or populism, intuitionism, the authoritarian personality, and, finally, romanticism. Within the literature on conspiracy thinking, there has been a small but growing attempt to circumvent the theoretical and conceptual problems outlined above by merely describing the empirical relationships among these measures as “anti-establishment orientations” or “populism” (Castanho Silva et al., 2017; Bergmann, 2018; Wood and Gray, 2019; Enders and Uscinski, 2021; Uscinski et al., 2021; Barker and DeTamble, 2022; Enders et al., 2022). Largely, these attempts are a-theoretical: they argue that the correlation among these measures (or some combination of these and other items) and authoritarian or extremist politics must be a unique construct because it is orthogonal to a left–right ideological dimension. While our own study shows that this is largely the case, an advancement in our understanding of the effects and correlates of this “anti-establishment” dimension requires a conceptual grounding missing in these studies.

A theory of the shared correlation would need to be able to explain how this person’s beliefs in the Loch Ness Monster or that alien contact is being withheld from the public are related to anti-democratic beliefs. For example, consider the collective picture of recent empirical findings that demonstrate that someone may come to support conspiracy theories and anti-democratic and authoritarian beliefs because of a “narcissistic” personality (Yendell and Herbert, 2022), and their reliance on intuition at the expense of mainstream authorities (van der Linden et al., 2021; Risen and Roberts, 2022; Moscatelli et al., 2023), that overestimates their own self-worth and legitimacy, all while trying to avoid boredom (van Prooijen, 2022). The connection between these traits and attitudes still requires a theoretical bridge to anti-democratic politics.

One potential candidate to tie these findings together is to call this person an “intuitionist” (Oliver and Wood, 2018, 49). Here “intuitionism” is a loose label for those who are higher on a latent dimension that is derived from three different subscales than those studied in this paper. Indeed, “intuition” is likely related to our shared dimension, which is why we included it in our post-hoc analysis, but it is not identical to it. Finally, one other potential candidate may be Adorno et al.’s original articulation of the authoritarian personality. We are sympathetic to this description of the shared variable, but, as discussed below, the authoritarian personality originally arose from reflections on the romantic notions and epistemologies, and the *F*-scale was an attempt to measure these psychological antecedents (Adorno et al., 2019).

To remedy the lack of overarching theory in the study of conspiracy thinking (Goreis and Voracek, 2019; Pilch et al., 2023), we believe that researchers should return to operationalizing the findings in the diagnostic literature during Nazism’s rise and fall. As noted above, Adorno and many of his contemporaries, including the postwar generation of German and American political psychologists associated first with the Frankfurt School and later the Columbia-affiliated Institute for Social Research that produced the so-called “California *F*-Scale,” began studying authoritarianism through shared romantic concepts like conspiracy thinking, esotericism, pseudoscience, folk mythology, “political religion” and other constructs whose relatedness they affirmed empirically. These scholars reached beyond conspiracy thinking as a way of understanding the rise of authoritarianism because the Nazis themselves appealed to more when understanding their political rise to power: in 1941, the Nazi Party’s ideological czar, Alfred Rosenberg, observed “that many Germans, due to their proclivity for the *romantic* [emphasis added] and the mystical, indeed the occult, came to understand the success of National Socialism in this fashion” (Kurlander, 2017). Our empirical findings are reminiscent of their original theories.

Though our empirical evidence does not warrant definitive claims as to the identity of the shared factor, in reviewing this literature in the context of our own empirical research, we have found the concept of romanticism to be a promising explanation. Generally, the Romantic movement was an esthetic reaction to the Enlightenment’s fixation on individualism, mechanism, and uniformity (Lovejoy, 1941). The romantic objects to individualism by appealing to “the collective,” that the individual is first and foremost a member of a community; in opposition to the Enlightenment’s fixation on a mechanical understanding of the world, the romantics emphasized the importance of “organicism” and “dynamism,” prioritizing change and struggle; finally, in opposition to the Enlightenment’s fixation on uniformity—that what defines human beings as moral agents is our shared humanity—the romantics privileged “diversity” and especially the differences between cultures—the strange, the historical, and the unique as what is of value (Lovejoy, 1924, 1941, 1944, 1964). In short, romanticism helps demarcate the shared conceptual neighborhood within which conspiracy thinking might be situated vis-à-vis belief in pseudoscience and paranormal thinking, while also establishing a set of historically grounded hypotheses and expectations about the relationship between these ideas and anti-democratic attitudes.

Although it is beyond the scope of this paper to fully develop and validate a social psychological scale of romantic thinking, the recent empirical findings of others can help outline the contours of what such a concept would entail. Our findings are a first step in theoretically recasting these disparate findings into a coherent whole: Romanticism. Further research is needed to uncover the precise nature of this romantic dimension, though synthesizing and replicating the extant findings in the literature would be a fruitful beginning to empirically validating what has historically motivated the field since Adorno’s F-scale.

### Limitations

There are several additional limitations to the current study that need to be acknowledged. First, the generalizability of our findings is limited by the non-probability sampling strategy and small sample sizes observed for specific demographic groups. Second, our data were cross-sectional, which inhibited our ability to carry out statistical hypothesis tests regarding any causal directional claims. Third, there were a variety of psychometric limitations to the scales we employed, but these limitations are hardly unique to this study, given the popularity of the scales and the sheer number of publications proposing alternative factor structures (see Measures section above). Nevertheless, we employed measures of esthetic, rational, and experiential intuitions that, while face valid, lack large scale testing and validation. As such, we limited their use to post-hoc exploratory analyses and caution against generalizing those findings to other settings until such validation takes place. We also are cognizant that all scales require continual refinement and empirical validation, and our findings should be replicated with other scales in the future.

Fourth, hierarchical factor models are not the only statistical approach to testing relationships among conspiracist, paranormal, and pseudoscientific beliefs and their collective associations with authoritarian tendencies. From a measurement perspective, an equally viable alternative approach is the bifactor model, which is often preferable due to the ease in interpreting test results when analyzing shared variance among multiple correlated items and constructs as in the current study. But given that these parallel approaches are so analytically similar (and even directly related through the Schmid-Leiman transformation), we ultimately opted for the hierarchical factor framework because it more directly reflects our theoretical claims regarding romanticism as a higher order factor that animates conspiracist, paranormal, and pseudoscientific beliefs as well as the claims of theorists from the interwar period who regarded “political romanticism” as a socio-political mood that preceded and facilitated the rise of magical and conspiratorial thinking, and in turn, fascism. Nevertheless, the option to employ the bi-factor framework represented an opportunity to test how sensitive our findings were to the choice of statistical approach, so we repeated our analyses within the bifactor framework and found nearly identical results.

Fifth and finally, we suspect that what we are delineating here is a multi-stage process, and that moving from “romantic” thinking to (rightwing) authoritarianism and social dominance orientation is more nuanced. It likely begins with a particular cognitive framework or set of values associated with romanticism (esthetic-occasionalist, intuitive, and/or simply romantic with a small “*r*”), which then manifests in certain attitudes toward science, religion, and sociopolitical reality as captured by conspiracism, pseudoscientific and paranormal thinking. It is a combination of these two components, the cognitive framework and its manifestation in civil society, that constitutes “Romanticism.” Conversely, in terms of the political expressions of “Romanticism,” SDO and RWA are useful but insufficient measures, which do not capture the full range of concrete political characteristics historians, sociologists, and political scientists associate with interwar fascism or, frankly, the contemporary alt-right. Indeed, SDO and RWA seem better suited to *anticipate*, in a sociopolitical and ideological sense, rather than explicitly define or express the concrete political manifestation of (politico-ideological commitments historically associated with) fascism. For these reasons, we will continue to refine and expand our project on “romanticism,” examining the psychological antecedents and correlates that trace the contours of romanticism. But just as importantly, we plan to draw again on the empirical evidence from and theoretical literature on fascism in interwar Germany and Europe to refine and expand upon SDO/RWA in developing a more accurate scale for measuring anti-democratic political commitments.

## Conclusion

Though some may doubt whether conspiracy thinking has increased in the past few decades, there’s no denying the reemergence of the political salience and use of conspiracy theories by authoritarian or anti-democratic actors (Uscinski et al., 2022). But, while conspiracy beliefs are used and believed by those who hold anti-democratic beliefs, this paper demonstrated that conspiracy thinking is not a unique predictor of authoritarianism. Instead, a shared factor of conspiracy thinking, paranormal thinking, and beliefs in pseudoscience is the driver of anti-democratic beliefs—and it is a factor that is stronger than partisanship. Conspiracy theory researchers should focus, then, on the shared features that conspiracy thinking has with other epistemically unwarranted beliefs. Only after we have demarcated this shared conceptual neighborhood can we begin to understand what is unique about conspiracy thinking. Indeed, the failures of interventions in stopping the spread of misinformation and conspiracy theories points to a failure in properly diagnosing the source of these beliefs. Perhaps reframing interventions to address the latent romantic character of these beliefs is a more fruitful way to halt the spread of misinformation, conspiracy thinking, anti-science attitudes, and even global authoritarianism.

## Data availability statement

The raw data supporting the conclusions of this article will be made available by the authors, without undue reservation.

## Ethics statement

The studies involving humans were approved by Stetson University Institutional Review Board. The studies were conducted in accordance with the local legislation and institutional requirements. The participants provided their written informed consent to participate in this study.

## Author contributions

SS was chiefly responsible for the theoretical motivation for the paper, collecting the theoretical and empirical literature for the literature review, writing and finalizing the manuscript (except where otherwise noted) for submission of the article, and the revisions of the article in light of reviewer comments, and for other aspects of the paper not claimed by the other authors. RA was chiefly responsible for all statistical analyses. He also was chiefly responsible for writing the methods and results sections of the manuscript. He also provided copy editing of drafts of the document. EK was responsible for the historical accuracy, including finding and incorporating historical literature in the literature review. He also provided copy editing of the drafts of the document. JR was responsible for revising the document in light of reviewer concerns about the rigor of the philosophical argumentation. He also wrote many of the reviewer responses. He also provided copy editing of the drafts of the document. All authors contributed to the article and approved the submitted version.
